# Experiences and perceptions from non-internal medicine clinicians deployed to COVID-19 units

**DOI:** 10.1192/j.eurpsy.2021.1778

**Published:** 2021-08-13

**Authors:** J. Tauber, J. Tingley, S. Rabbanifar, A. Bitners, A. Shrivastava, C. Skae

**Affiliations:** 1 Ophthalmology, Montefiore Medical Center/Albert Einstein College of Medicine, Bronx, United States of America; 2 Psychiatry, Montefiore Medical Center/Albert Einstein College of Medicine, Bronx, United States of America; 3 Medical School, Albert Einstein College of Medicine, Bronx, United States of America; 4 Graduate Medical Education, Montefiore Medical Center/Albert Einstein College of Medicine, Bronx, United States of America

**Keywords:** deployment, preparedness, COVID-19, stress

## Abstract

**Introduction:**

When New York City became an epicenter of the COVID-19 pandemic, healthcare workers from an array of specialties were deployed to work on general medicine units with limited time for clinical retraining.

**Objectives:**

This study assesses the subjective experience and perceived preparedness of a cohort of non-internal medicine clinicians who were deployed to assist with inpatient management of patients with COVID-19 in the Spring of 2020.

**Methods:**

An online survey was distributed to clinicians (residents, fellows, attendings, nurse practitioners, and physician assistants) who cared for patients in roles outside their usual specialties during the pandemic at the Montefiore Health System in the Bronx, NY.

**Results:**

85/169 (50.3%) clinicians responded. 16.5% reported strong feelings of preparedness prior to deployment (≥7/10 Likert scale). ‘Access to appropriate and efficient review materials prior to deployment’ was ranked as 6/10, overall level of stress as 8/10 and concern for contracting COVID-19 while deployed as 8/10. Responses regarding ‘general feelings of preparedness’ had a weak negative association with ‘feelings of frustration about one’s circumstance’ (r= -0.39, p<0.001). Weak negative associations were found between feelings of ‘access to adequate review materials’ and ‘overall stress levels’ (r= -0.31, p<0.001). A moderate positive association was found between ‘feelings of access to adequate review materials’ and ‘feeling on top of one’s work responsibilities’ (r= 0.40, p< 0.001).

**Conclusions:**

The majority of respondents did not feel adequately prepared to care for patients with COVID-19 prior to deployment and had both high stress levels and fear of contracting COVID-19 in the first wave of the pandemic.

**Disclosure:**

No significant relationships.

